# Exploring long-term cancer survivors’ care experiences and unmet needs: protocol for a qualitative study

**DOI:** 10.1186/s12885-024-12527-9

**Published:** 2024-07-01

**Authors:** Christian Speckemeier, K Maus, A Bialobrzeski, B Jaspers, L Radbruch, S Hahn, J Wasem, V Grünwald, U Dirksen, A Neumann

**Affiliations:** 1 Institute for Healthcare Management and Research, University of Duisburg-Essen, 45127 Thea-Leymann-Str. 9, Essen, Germany; 2 Department of Palliative Medicine, University Hospital Bonn, 53127 Venusberg-Campus 1, Bonn, Germany; 3 Clinic for Urology, Clinic for Medical Oncology, University Hospital Essen, 45147 Hufelandstr. 55, Essen, Germany; 4 Paediatrics III, German Cancer Research Centre (DKTK), West German Cancer Center, National Center for Tumordiseases (NCT) site Essen, University Hospital Essen, 45147 Hufelandstraße 55, Essen, Germany; 5 Department of Pediatric Hematology and Oncology, University Hospital Bonn, 53127 Venusberg-Campus 1, Bonn, Germany

**Keywords:** Cancer survivorship, Long-term, Minority, Interview, Focus group, LGBT

## Abstract

**Background:**

The number of cancer survivors has increased in recent decades, and the majority of them suffer from sequelae of their disease and treatment. This study, which is part of the larger research project OPTILATER, aims to explore different aspects of care services for long-term survivors (≥ 5 years after initial cancer diagnosis) in Germany. The study places an emphasis on the situation of people from different age groups, with different socio-demographic and cultural backgrounds, and sexually and gender diverse individuals.

**Methods:**

To investigate experiences related to follow-up care, focus groups (*n* = 2) will be conducted with members of patient advisory councils and advocacy groups, representatives of communities, healthcare workers and networks, as well as members of Associations of Statutory Health Insurance Physicians. Guided interviews will be carried out with patients and relatives (n = 40) to investigate needs, barriers and obstacles in terms of follow-up care. On this basis, additional focus groups (*n* = 2) will be carried out to derive possible scenarios for improving the consideration of needs. Focus groups and interviews will follow a semi-structured format and will be analysed content-analytically. Focus groups and interviews will be conducted online, recorded, transcribed, and analysed independently by two persons.

**Discussion:**

The qualitative approach is considered suitable because of the exploratory research aims. The identification of experiences and barriers can reveal disparities and optimization potential in the care of long-term cancer survivors.

## Introduction

Nearly 500,000 new cases of cancer are diagnosed in Germany every year. By 2030, this number is expected to increase by 23% compared to 2015, which is mainly due to demographic developments [1]. According to estimates, around 4.5 million people in Germany are currently living with or after a cancer diagnosis and of these, about 2.6 million have survived the cancer diagnosis five or more years [2].

Owing to advancements in cancer detection and treatment, the number of cancer survivors has increased considerably over the last decades [3]. Only a small proportion of survivors live without related problems, while most continue to suffer from the consequences of the disease and aggressive treatment regimens even after active treatment has been completed [4]. Cancer survivors experience a variety of physical, emotional, and social problems [5]. They frequently suffer from fears regarding their future, sleep difficulties, depression, fatigue, loss of strength, neuropathy, pain, osteoporosis, diabetes, heart failure, and cognitive impairment, among others [5–9]. Some of these symptoms may persist for an extended time after treatment has ended and become long-term sequelae [5], which are known to significantly impair quality of life [10]. In addition, survivors may also experience a range of financial, economic, and employment problems [9]. Recently, many of the issues experienced by cancer survivors have been exacerbated by the COVID-19 pandemic and the associated contact restrictions and isolation measures [11].

In Germany, knowledge gaps have been identified regarding the situation of persons living with and after cancer [12]. According to the German Ministry of Health, one focus of research should be on the targeted collection and evaluation of data on the needs, requirements and quality of life of cancer survivors and their families [2]. In addition, efforts are being made to learn something from the group of cancer survivors with reference to resilience as an object of research. In this regard, the term “post-traumatic growth” has long been discussed in the psychological context [13–15]. While a variety of support and information resources for cancer survivors exists in Germany, it is currently unclear as to whether these are used extensively and, even more, if they succeed in addressing people from different age groups, with different sociodemographic and cultural backgrounds, as well as sexually and gender diverse individuals. In palliative care research, there are already some efforts to raise awareness or even improve researchers’ consideration of underrepresented groups [16].

International literature indicates inequality in care for cancer survivors [17, 18]. For example, disparities in relation to race/ethnicity and/or sexual orientation were found with respect to post-treatment use [19] and physical and mental health outcomes [20, 21]. Sexual and gender diverse cancer survivors often experience negative healthcare encounters, face discrimination, and are more likely to experience depression when compared to heterosexual cancer survivors [18]. Barriers to culturally competent care faced by sexual and gender minorities thus lead to worse patient experiences and health outcomes [22]. For persons with a migrant background and individuals belonging to ethnic minorities, language and cultural factors are barriers that complicate all areas of information needs [23]. Moreover, a systematic review has found a lower healthcare utilization among individuals with a migration background in Germany, with lower usage being particularly pronounced among children/adolescents, women, persons with two-sided migration background, and migrants of the first generation [24]. Currently, around 29% of the population in Germany (23.8 million people) have a migrant background [25], which makes these issues a topic of high social relevance and of importance for daily practice in oncology [23].

Identification of the barriers faced by minorities can highlight areas of exclusion and inequality, with the aim to dissolve these structures. The project OPTILATER (Optimal long-term survival after cancer) [26], which is funded by the German Ministry of Health, seeks to analyse and provide recommendations for improved care of long-term cancer survivors in the federal state of North Rhine-Westphalia in Germany. In OPTILATER, long-term cancer survivors are defined as people who have survived longer than five years after diagnosis of cancer. The project consists of seven work packages, including an analysis of the current situation based on claims and registry data, qualitative research, and literature reviews, as well as the derivation of the desired situation and formulation of evidence-based recommendations. Qualitative work pertaining to work package (WP) 2, which is covered in this research protocol, includes the conduct of focus groups and semi-structured interviews. WP2 aims to explore (i) the experiences of cancer survivors (≥ 18 years of age at the time of the study) and significant others (persons who have a very close and special relationship to the patient, e.g. parents, friends, etc.) as well as health care professionals with care services (ii) unmet or insufficiently met needs of patients/significant others as well as barriers and obstacles to utilization, and (iii) possible scenarios for improving the consideration of needs. For all these aspects, the impact on quality of life and possible implications through facets of gender/diversity, socioeconomic status, migration background and age are considered. Moreover, the time of diagnosis is taken into account. In addition, information and counselling needs, interests, preferences and behaviours, possible barriers to access and use of information as well as counselling services are addressed throughout the process.

## Methods

**Fig. 1:**
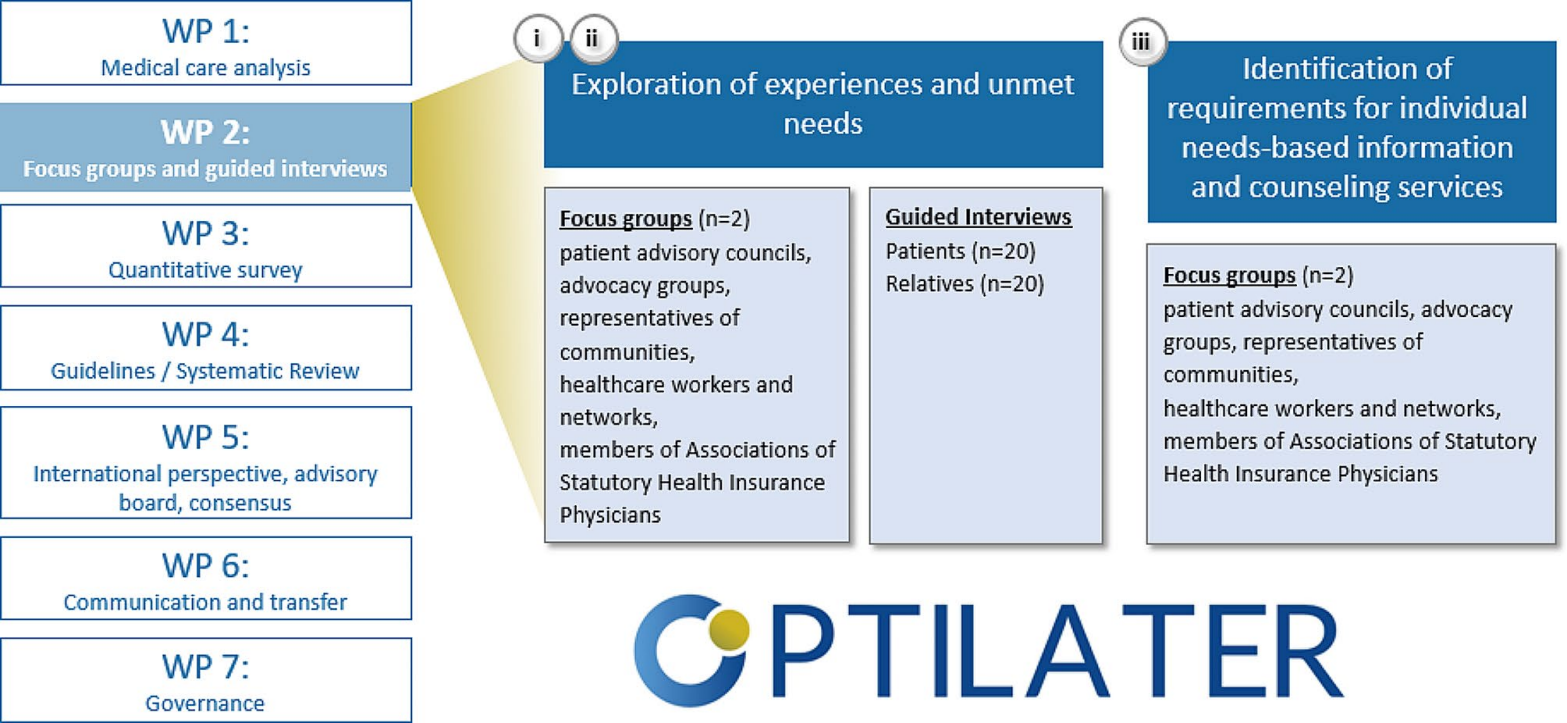
Overview of the qualitative research in WP2 as part of the OPTILATER project WP, work package.

The study was designed and will be carried out according to the qualitative research guidelines (SRQR guidelines) [27]. An overview of the qualitative research is shown in Fig. 1.

OPTILATER is characterized by a strong networking character between the WPs, with WP2 being interlocked with other WPs at several points. For example, information on the supply situation with regards to existing consulting and communication structures (WP1) will serve as a basis for the interview guides in WP2. Likewise, the results of the literature review on guidelines (WP4) will serve as a basis for the interview guides in WP2. As outlined below, the target sample of the quantitative survey (WP3) will be asked for their willingness to be interviewed in WP2. Raw results of WP2 will serve as basis for a secondary analysis in WP3.

### Sample

Initially, two focus groups with members of patient advisory councils and advocacy groups, representatives of communities, healthcare workers and networks, as well as members of Associations of Statutory Health Insurance Physicians will be conducted. Based on this, a total of 40 guided interviews with patients and significant others will be conducted, taking into account aspects of gender/diversity, socioeconomic status, migration background, etc. Patients and significant others are included if the cancer was diagnosed at least five years ago. Research aim (iii) seeks to identify requirements for individual needs-based information and counselling services as well as accessibility to these services. These aspects will be explored via two focus groups consisting of the same participants as the focus groups recruited for (i) and (ii).

### Recruitment

Approval to conduct the focus groups and guided interviews has been obtained by the Ethics Committees of the institutions involved. Participants will be recruited from patient organisations, participating health insurances, resident Associations of Statutory Health Insurance Physicians, and communities. If possible, members of the focus groups pertaining to research aim (iii) should be the same persons which were recruited for (i) and (ii). For the guided interviews, the target sample of the quantitative survey (WP3) will be asked for their basic willingness to be interviewed. If willing, respondents can get in touch with WP2 researchers for detailed information. Furthermore, self-help groups, communities etc. will be contacted. Potential participants receive a letter containing information on data protection and are asked for their consent to participate. Participants will be informed about the purpose of the study, the research objective, and the handling of their data. Participants will also be informed that for further scientific analysis, all information that could lead to an identification of the person will be changed or removed from the text. For this purpose, the identifying characteristics will be pseudonymised.

### Development of interview guides

In collaboration with the clinicians in the project consortium, a semi-structured and target-group specific guidance will be developed for the focus groups and interviews. A systematic approach (SPSS-method) will be followed. Open guiding questions, maintenance questions, and more in-depth follow-up questions will first be collected (S), reviewed (P), sorted (S), and finally subsumed (S) [28]. The guide will be piloted before the start of the interview phase among three to five patients and significant others who will not be participants. Probing and think-aloud will be used to identify potential problems in comprehensibility and feasibility and the guidance will be adapted accordingly.

### Data processing and analysis

Focus groups and interviews will be conducted online, recorded via a videoconferencing system, and subsequently put into written form. Socioeconomic data will be collected in a separate online survey. After each focus group, field notes will be documented immediately. Participants’ roles will be described and verbal and nonverbal communication styles recorded. In the evaluation of the focus groups, the thematic framework will first be identified and the information will be summarized for evaluation based on word choice, context, internal consistency, frequency and scope of themes, intensity and specificity, and broad contexts. The Constant Comparative Method [29] will be used to capture saturation in the evaluation from the four focus groups individually and across groups. Specific themes from each focus group are compared across all groups.

Interviews will be transcribed verbatim and analysed using MAXQDA 2022 (Verbi Software GmbH, Berlin). The evaluation of the interviews is carried out content-analytically according to Kuckartz [30], while taking into account the formulated objectives of WP2 with a focus on inequalities. The evaluations of two individual interviews are first carried out independently by two researchers. Afterwards, the coding and the codebook will be discussed, aligned and harmonized. Further, the analysis of the individual interviews will be split and iteratively validated. In case of incongruence, a third researcher will be consulted for discussion. Following this analysis, all material will be reviewed one more time with regard to intersectionality aspects in order to explore the interrelationships of different forms and dimensions of diversity in the material at hand.

## Discussion

The project OPTILATER aims to identify new pathways in the care of long-term cancer survivors in order to develop a diversity and culturally sensitive program, and ultimately to improve clinical outcomes and quality of life. OPTILATER focuses on cancer survivors whose diagnosis dates back at least five years. While cancer survivorship is commonly defined as “the experience of living with, through and beyond a diagnosis of cancer” [31] and thus includes patients whose primary treatment has not yet been completed, the study focuses on long-term survivors. These survivors will have completed their primary treatment or major aspects of it and ‘continue with their lives’ [32]. While acute cancer treatment is usually clearly outlined, this is not true to the same extent for the treatment of long-term survivors [3]. Due to complex health care structures, cancer survivors need to take an active role in their healthcare [33]. At the same time, quality of life is impaired in a large proportion of long-term survivors, resulting in the desire for improvements in long-term follow-up care [34].

The complexity of care structures and evident barriers of certain minorities, combined with the relevance to society as a whole, make it important to investigate care for long-term cancer survivors. An adequate and needs-oriented support could be described as ‘a key challenge for health care professionals caring for this population’ [35]. For this purpose, the involvement of those affected is also necessary. The project aims will be achieved through explorative research, namely focus groups and interviews, as outlined in this protocol. To allow for a comprehensive analysis, a total of at least 20 people should be involved in the four focus groups and 40 people in the individual interviews. Participants comprise patients, relatives and diverse stakeholders. This approach is in line with the NCCS definition of cancer survivorship cited above, which explicitly includes family members and friends affected by the experience. Based on the experiences, needs, barriers and obstacles identified via focus groups and interviews, a second series of focus groups will be undertaken to derive possible scenarios for improving the consideration of needs. Our methodological approach thus aims to develop recommendations for action with potentials for improving the information and care of long-term cancer survivors, taking into account the special needs of minorities. With this, we follow the hypothesis that “intersectionality informed research transcends the mere description of health inequities and focuses on the goal of social justice as a mechanism for social change and transformation” [36].

## Data Availability

No datasets were generated or analysed during the current study.
